# Enhancing the Electromagnetic Interference Shielding Effectiveness of a AZ61 Magnesium Alloy by Deformation and Subsequent Heat Treatment

**DOI:** 10.3390/ma19071383

**Published:** 2026-03-31

**Authors:** Minhyeok Kang, Kyengtaek Kim, Seongje Kim, Jose Victoria-Hernandez, Dietmar Letzig, Sangbong Yi

**Affiliations:** 1 School of Materials Science and Engineering, Kumoh National Institute of Technology, Gumi 39177, Republic of Korea; lkoid12@kumoh.ac.kr (M.K.); 20190093@kumoh.ac.kr (K.K.); sje8448@kumoh.ac.kr (S.K.); 2 Helmholtz-Zentrum Hereon, Institute of Material and Process Design, 21502 Geesthacht, Germany; dietmar.letzig@hereon.de

**Keywords:** electromagnetic interference shielding, rolling, aging

## Abstract

**Highlights:**

- The increased dislocation density and deformation twins induced by hot rolling contribute to enhanced electromagnetic interference (EMI) shielding effectiveness.
- During aging of the AZ61 alloy, precipitates preferentially form along the basal (0002) planes.
- The increased density of precipitates distributed along the basal (0002) planes contributes to improved electromagnetic interference (EMI) shielding effectiveness.
- This study provides fundamental insight into the mechanisms underlying the electromagnetic interference (EMI) shielding effectiveness of the hot-rolled and aged AZ61 alloy.

**Abstract:**

The rapid advancement and widespread application of telecommunication technologies have significantly increased human exposure to electromagnetic waves, thereby intensifying the demand for effective electromagnetic shielding materials. Beyond potential health concerns, ensuring the stable performance of highly integrated electronic devices also necessitates protection against electromagnetic interference (EMI). In this study, the effects of processing conditions on the EMI shielding effectiveness (SE) of AZ61 magnesium alloy sheets were systematically investigated. Aging treatment of rolled AZ61 alloy promoted the formation of Mg_17_Al_12_ lamellae. Transmission Kikuchi diffraction analysis revealed that plate-like Mg_17_Al_12_ precipitates preferentially formed on the (0001) planes of the Mg matrix, contributing to improved EMI shielding. The rolled AZ61 sheet exhibited the highest SE in both the as-rolled state (83.1 dB at 900 MHz) and after aging for 131 h at 250 °C (76.2 dB at 900 MHz). The superior shielding performance of the as-rolled sheet is attributed to its high density of deformation-induced defects such as dislocations and twins, which induce lattice distortions and impede wave propagation. Meanwhile, the enhanced SE from the 131 h-aged condition results from multiple reflections of incident electromagnetic waves facilitated by the matrix–precipitate lamellar microstructure.

## 1. Introduction

Recent advances in electronic devices and wireless communication technologies have raised increasing concerns regarding electromagnetic interference (EMI), attracting significant attention from both academia and industry. EMI is known to induce malfunctions and unexpected failures in electronic devices under service conditions [1,2,3]. Consequently, effective EMI shielding is essential to ensure the reliable performance and durability of modern electronic systems.

The shielding effectiveness (SE), expressed in decibels (dB), is a key parameter for quantifying the EMI shielding capability of a material. As an electromagnetic wave interacts with a shielding medium, three primary mechanisms contribute to its attenuation: reflection, absorption, and multiple reflections. The total shielding effectiveness (${SE}_{tot}$) is expressed as [4]:

$${SE}_{tot}={SE}_{R}+{SE}_{A}+{SE}_{B}$$

where ${SE}_{R}$, ${SE}_{A}$ and ${SE}_{B}$ represent the contributions from reflection, absorption, and multiple reflection, respectively. ${SE}_{R}$, ${SE}_{A}$ and ${SE}_{B}$ are defined as follows [4].

$${SE}_{R}=168-10\log({f\mu }_{r}/\sigma_{r})\;,$$

$${SE}_{A}=1.314t{\left(f\mu_{r}\sigma_{r}\right)}^{\left(1/2\right)}\;,$$

$${SE}_{B}=20\log\left(1-\left(e^{2t/\delta }\right)\right)\;,$$

$$\delta ={\left(\pi \mu \sigma f\right)}^{(-1/2)}\;,$$

$f,\mu_{r},\mu ,\sigma_{r},\sigma$ and t denote the frequency of electromagnetic radiation, relative magnetic permeability, magnetic permeability, relative electrical conductivity, electrical conductivity, and shield thickness, respectively. For conductive materials such as metals, the majority of incident electromagnetic waves are reflected at the surface, while only a small fraction penetrates into the material. As a result, attenuation by reflection (${SE}_{R}$) generally provides the dominant contribution to ${SE}_{tot}$, with absorption and multiple reflection further attenuating the transmitted waves.

Highly conductive metals such as Cu and Ag exhibit excellent EMI shielding performance. However, their high density limits their applicability in fields such as transportation and telecommunications. Accordingly, the development of lightweight materials with superior EMI shielding capabilities has attracted growing research interest over the past decade.

Magnesium and its alloys have emerged as promising candidates due to their unique combination of properties, including the lowest density among structural metals, high specific strength, excellent damping capacity, and favorable electrical conductivity [5,6,7]. The EMI shielding effectiveness of magnesium alloys has been shown to depend on alloying elements, crystallographic texture, and the formation of secondary phases [8]. For example, Song et al. [9] reported that strengthening of the basal-type texture enhances the SE of AZ31 alloy. Chen et al. [10] demonstrated that the orientation of precipitates in Mg–3Sn, Mg-5Sn alloys significantly affects EMI shielding, with improved SE observed when precipitates align along the RD–TD plane, i.e., when the incident electromagnetic wave is perpendicular to the basal plane. These studies highlight the importance of microstructural features such as precipitate morphology, crystallographic texture, and grain size in governing the EMI shielding performance of Mg alloys.

In the present work, the microstructural evolution of Mg–Al–Zn alloy subjected to hot rolling and subsequent aging treatment was systematically characterized. Particular attention was given to the effects of deformation-induced microstructural features and secondary-phase precipitation on both the shielding effectiveness and the mechanical properties.

## 2. Materials and Methods

Commercial AZ61 alloy ingots were homogenized at 350 °C for 15 h. Subsequently, rectangular billets with a cross-sectional area of 12 × 93 mm^2^ were fabricated by direct extrusion at 250 °C, employing a constant ram speed of 10 mm·s^−1^ and an extrusion ratio of 10. The extruded billets were then hot-rolled at 400 °C through 10 passes with a pass reduction of 15–18%, resulting in a final sheet thickness of 1 mm. Solution treatment was performed on the rolled sheets at 400 °C for 18 h. Aging heat treatment was conducted at 250 °C for up to 262 h. The specimens corresponding to different processing conditions were categorized as summarized in Table 1.

Samples for microstructural analysis were prepared by sequential grinding up to #4000 emery paper, followed by mechanical polishing with a colloidal silica suspension. The polished specimens were subsequently etched in a solution consisting of 4.2 g picric acid, 10 mL distilled water, 10 mL acetic acid, and 70 mL ethanol for 1–5 s. Microstructural observations were conducted using an optical microscope (OM, BX53M, Olympus, Tokyo, Japan) and a scanning electron microscope (SEM, 700HR, JEOL, Tokyo, Japan) equipped with energy-dispersive X-ray spectroscopy (EDX). Electron backscatter diffraction (EBSD) combined with focused ion beam (FIB) milling was employed to characterize the crystallographic orientation relationships between the secondary phases and the matrix grains. X-ray diffraction (XRD, Miniflex 600, Rigaku, Tokyo, Japan) was employed to analyze the crystallographic orientation and to identify the phases formed under various conditions. Mechanical properties were evaluated by using a Vickers hardness tester (VMT-X7, Matsuzawa, Akita, Japan) under an applied load of 1 kgf for 10 s.

**Figure 1:**
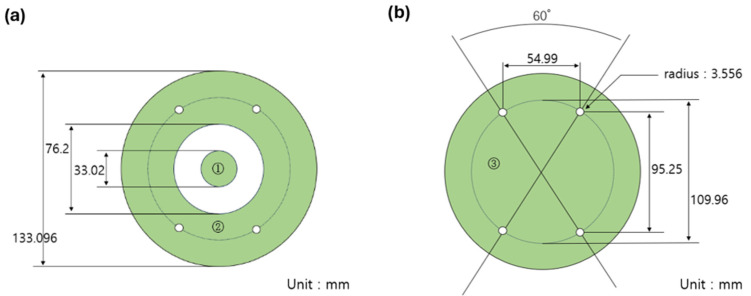
Dimensions of specimens (in mm) for EMI SE measurements in accordance with ASTM D4935: (**a**) base specimen and (**b**) load specimen.

Specimens for electromagnetic interference shielding effectiveness (EMI SE) measurements were fabricated by spark erosion in accordance with ASTM D4935 [11], as illustrated in Figure 1. Two types of specimens were prepared: the base specimen, Figure 1a and the load specimen, Figure 1b. EMI SE was measured using a network analyzer (ZVA40, Rohde & Schwarz, Munich, Germany) over a frequency range of 30 MHz to 1.5 GHz. The base specimen was measured first, followed by the load specimen. The EMI SE of the material was determined by subtracting the measurement obtained for the load specimen from that of the base specimen.

## 3. Results and Discussion

**Figure 2:**
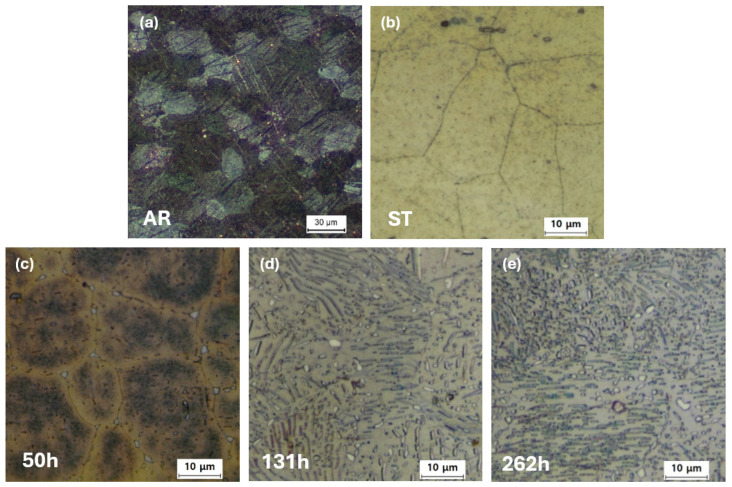
Optical micrographs of the AZ61 alloy under different heat-treatment conditions: (**a**) as-received (AR), (**b**) solution-treated (ST), (**c**) aged for 50 h (50 h), (**d**) aged for 131 h (131 h), and (**e**) aged for 262 h (262 h).

Optical micrographs of the samples subjected to various heat treatments are presented in Figure 2. All observations were performed on the RD–ND plane of the sheet specimens. The as-rolled (AR) sample exhibits a high density of deformation twins with an average grain size of 20–40 µm (Figure 2a). Following solution treatment (ST) at 400 °C for 18 h, the grain size remains essentially unchanged, confirming that grain coarsening is effectively suppressed during the treatment (Figure 2b). This stability is attributed to the grain boundary pinning effect of fine precipitates in the AZ61 alloy [12]. With subsequent aging treatments, the amount of precipitates increases progressively, as shown in Figure 2c–e. Especially, lamellar precipitates were observed within the Mg matrix after 131 h of aging (Figure 2d,e). The precipitate length ranged from <1 µm to over 20 µm on the observed surface.

**Figure 3:**
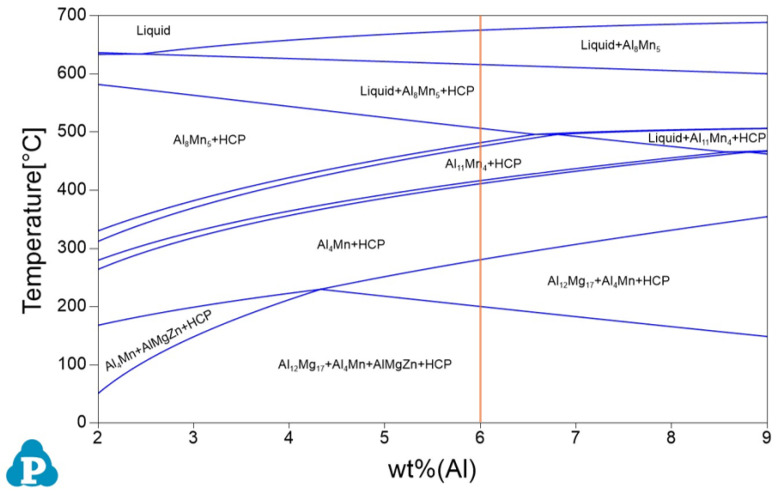
Phase diagram of the Mg–1Zn–0.4Mn–(2–9)Al (wt.%) alloy system, simulated with the thermodynamic software Pandat-2025.

The calculated phase diagram of the (2-9)Al–1Zn–0.2Mn–Mg alloy with Al contents ranging from 2 to 9 wt.% is shown in Figure 3, obtained by using the thermodynamic simulation software Pandat-2025. A guideline is included to indicate the composition of the present AZ61 alloy (Figure 3). The phase diagram predicts the formation of two types of precipitates in this alloy system: Al–Mn phases and Al–Mg phases. The Al–Mg phases are thermally unstable and dissolve during solution heat treatment at 400 °C, whereas the Al–Mn phases remain stable and do not dissolve at this temperature. Consequently, the precipitates observed in the solution-treated (ST) specimen are mainly the Al–Mn phases, which suppressed grain coarsening by exerting a grain boundary pinning effect. With subsequent aging, the number density and size of precipitates continuously increased, as shown in Figure 2c–e. These precipitates were identified as Mg_17_Al_12_ phases, consistent with the phase diagram.

**Figure 4:**
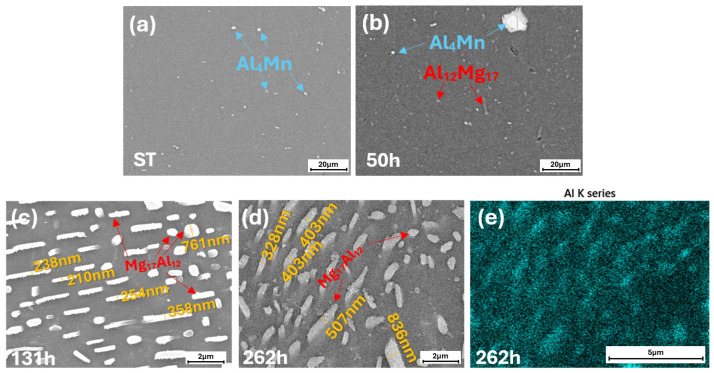
SEM micrographs and EDX mapping of AZ61 alloy. (**a**) SEM image of ST specimen, (**b**) 50 h, (**c**) 131 h, (**d**) 262 h, (**e**) aluminum EDX mapping image 262 h specimen.

The chemical compositions of the precipitates were analyzed by SEM–EDX analysis, Figure 4. All SEM micrographs were acquired at the RD–ND plane. SEM–EDX analysis of the ST specimen confirmed the persistence of Al–Mn phases, Figure 4a. Upon subsequent aging heat treatment, Mg_17_Al_12_ precipitates is identified, as exemplified in the 50 h specimen, Figure 4b, consistent with the phase diagram in Figure 3. With prolonged aging, the volume fraction of Mg_17_Al_12_ increased, Figure 4c. A comparison between Figure 4c,d indicates that the 262 h specimen contained coarser Mg_17_Al_12_ precipitates than the 131 h specimen. The thickness of the Mg_17_Al_12_ precipitates in the 131 h specimen ranged from 210 to 761 nm, whereas those in the 262 h specimen ranged from 328 to 836 nm. The EDX mapping corresponding to the area of Figure 4d,e indicated a high Al content within the precipitates, confirming that they are Mg_17_Al_12_ precipitates.

**Figure 5:**
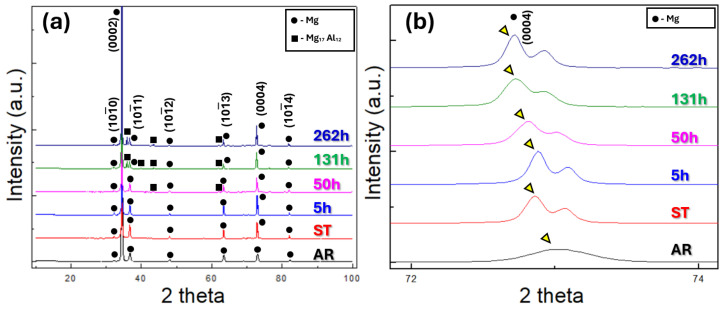
XRD patterns of the AZ61 alloy: (**a**) wide scan (2θ = 10–100°) and (**b**) magnified region (2θ = 72–74°).

Figure 5 presents the XRD diffraction profiles of the examined specimens. All specimens exhibit a strong (0002) diffraction peak, which can be attributed to the crystallographic texture developed during the hot rolling process. The XRD patterns of the AR and ST specimens exhibit only Mg peaks, without the diffraction peaks corresponding to secondary phases. In the 5 h specimen, diffraction peaks corresponding to the Mg_17_Al_12_ phase appear as a result of the aging treatment. As the aging time increases from 5 h to 262 h, the intensity of the Mg_17_Al_12_ peaks progressively increases, confirming the continuous precipitation and growth of Mg_17_Al_12_ in the aged specimens.

Figure 5b presents an enlarged view of the XRD profiles at high 2θ range. A comparison of the (0004) peak positions, indicated by yellow triangles, reveals distinct differences among the specimens. The AR specimen exhibits its (0004) peak at the rightmost position compared to the others. This shift can be attributed to compressive residual stresses introduced during hot rolling. Such stresses reduce the lattice constant, and according to Bragg’s law (nλ = 2dsinθ), a decreased lattice constant results in a peak shift toward higher 2θ values.

Among the remaining specimens, the solution-treated and 5 h-aged samples also show their (0004) peaks located at the rightmost side. During solution treatment of AZ61 alloy, Al atoms dissolve into the Mg matrix to form a solid solution. Since the atomic radius of Al is smaller than that of Mg, the lattice constant of the Mg matrix decreases, leading to a peak shift to higher 2θ values in accordance with Bragg’s law. Upon subsequent aging, these Al solutes precipitate as Mg_17_Al_12_, allowing the Mg matrix to recover its intrinsic lattice parameter, which is larger than that of the Al-containing Mg solid solution. Consequently, the diffraction peak shifts back toward lower 2θ values. Nevertheless, after 5 h of aging, a considerable amount of Al solutes still remains in the Mg matrix, comparable to that of the solution-treated state. As a result, the (0004) peak of the 5 h aged specimen also remains at relatively high 2θ values, consistent with the observed XRD results in Figure 5b. Thus, the precipitation of the Mg_17_Al_12_ phase can also be observed through the shift in the (0004) diffraction peak.

**Figure 6:**
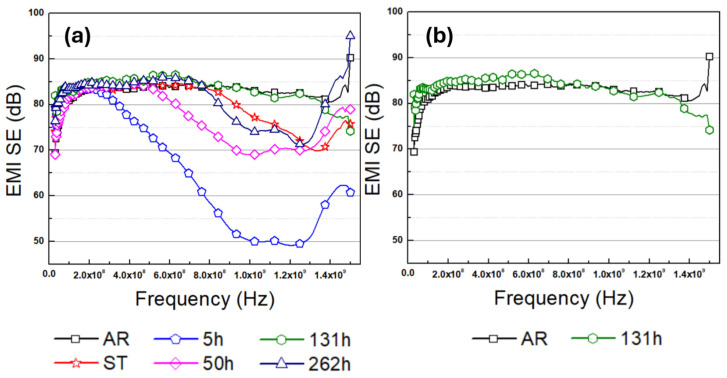
Electromagnetic interference (EMI) shielding effectiveness of AZ61 specimens measured in the frequency range of 30 MHz–1.5 GHz. (**a**) Results for all investigated specimens (**b**) Comparison limited to the two specimens exhibiting the highest EMI SE values.

The EMI SE of the examined specimens is demonstrated in Figure 6. As shown in Figure 6a, the AR and 131 h specimens exhibit the highest EMI SE values among the examined AZ61 samples. The electromagnetic interference (EMI) shielding effectiveness (SE) values of the AZ61 specimens at different incident frequencies are summarized in Table 2. Among the investigated samples, the 131 h-aged specimen exhibited the highest SE of 77.8 dB, which is approximately 32.5% greater than that of the 5 h-aged specimen. The superior EMI shielding performance of the AR and 131 h-aged specimens can be attributed to diverse mechanisms.

Table 3 presents the electrical conductivity of AZ61 alloy samples subjected to different processing conditions. The as-rolled (AR) sample exhibits the lowest electrical conductivity, which can be attributed to the high density of dislocations introduced during the rolling process. The solution-treated (ST) sample shows a slightly higher conductivity than the AR sample. Solution heat treatment reduces lattice defects such as dislocations, thereby improving electrical conductivity. Although solution heat treatment increases the concentration of solute atoms, particularly Al, in the Mg matrix—which generally leads to a reduction in electrical conductivity—this effect appears to be offset by the decrease in defect density during the solution treatment. Furthermore, the electrical conductivity increases with increasing aging time when comparing the aged samples with the ST condition. This improvement can be attributed to the precipitation of Mg_17_Al_12_ phases during aging, which reduces the concentration of solute atoms in the Mg matrix and consequently enhances electrical conductivity.

It is noteworthy that the as-rolled (AR) specimen exhibits the lowest electrical conductivity while demonstrating the highest EMI shielding effectiveness (EMI SE). As described in the introduction, a reduction in electrical conductivity generally decreases EMI SE by reducing the reflection component (${SE}_{R}$), which is the dominant contributor to the shielding performance of metallic materials. Therefore, the observed high EMI SE cannot be explained solely by electrical conductivity. Instead, the results suggest that microstructural factors introduced during the hot-rolling process play a significant role in enhancing the shielding effectiveness of the AZ61 alloy.

Firstly, defects such as grain boundaries and secondary phases can attenuate incident electromagnetic waves by reflecting the incoming radiation [8,13]. In addition, the superior EMI SE of the AR specimen, which contains a high density of dislocations and deformation twins introduced during hot rolling, indicates that dislocations and twins also play a significant role in the attenuation of electromagnetic waves. When comparing the AR specimen with its high dislocation density and a lot of dislocation twins to the ST specimen which has relatively low dislocation density and dislocation twins, the former possesses a more complex atomic arrangement. Such structural complexity increases the probability of collisions between the penetrating electromagnetic waves and lattice atoms, thereby reducing the mean free path of the radiation. Conversely, in the matrix with lower deformation induced defects such as dislocation and twins, electromagnetic waves experience longer mean free paths, resulting in reduced attenuation. Consequently, the AR specimen exhibits excellent EMI SE, whereas the shielding performance of the ST specimen and the aged specimens up to 50 h is diminished.

**Figure 7:**
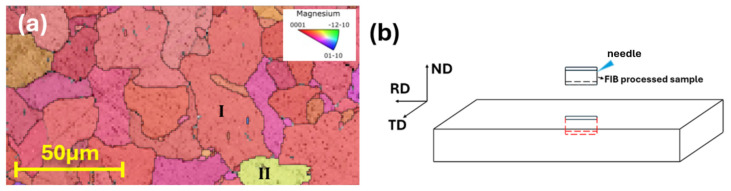
(**a**) EBSD inverse pole figure (IPF) map of the 131h specimen Region I (denoted as 131h-I) corresponds to grains exhibiting basal orientation, whereas Region II (131h-II) represents regions with comparatively non-basal orientation. (**b**) Schematic illustration of the FIB-based specimen extraction procedure from the 131 h specimen.

To examine the microstructural characteristics of the 131 h-aged specimen, electron backscatter diffraction (EBSD) analysis was performed on slice samples sectioned along the RD–TD plane. Figure 7a shows the EBSD inverse pole figure (IPF) map of the 131 h specimen. A pronounced basal texture was observed, which is consistent with the XRD results, where the (0002) diffraction peak exhibited strong intensity, Figure 5a. To obtain detailed information on the orientation of the Mg_17_Al_12_ precipitates, two FIB slices were extracted from the RD–TD plane along the ND. The orientation of the FIB slices with respect to the sample geometry is schematically illustrated in Figure 7b. One slice was prepared from a grain with its crystallographic c-axis aligned parallel to the ND (grain I in Figure 7a), hereafter referred to as 131h-I. The other slice was taken from a grain with the c-axis tilted by approximately 52° from the ND (grain II in Figure 7a), denoted as 131h-II.

**Figure 8:**
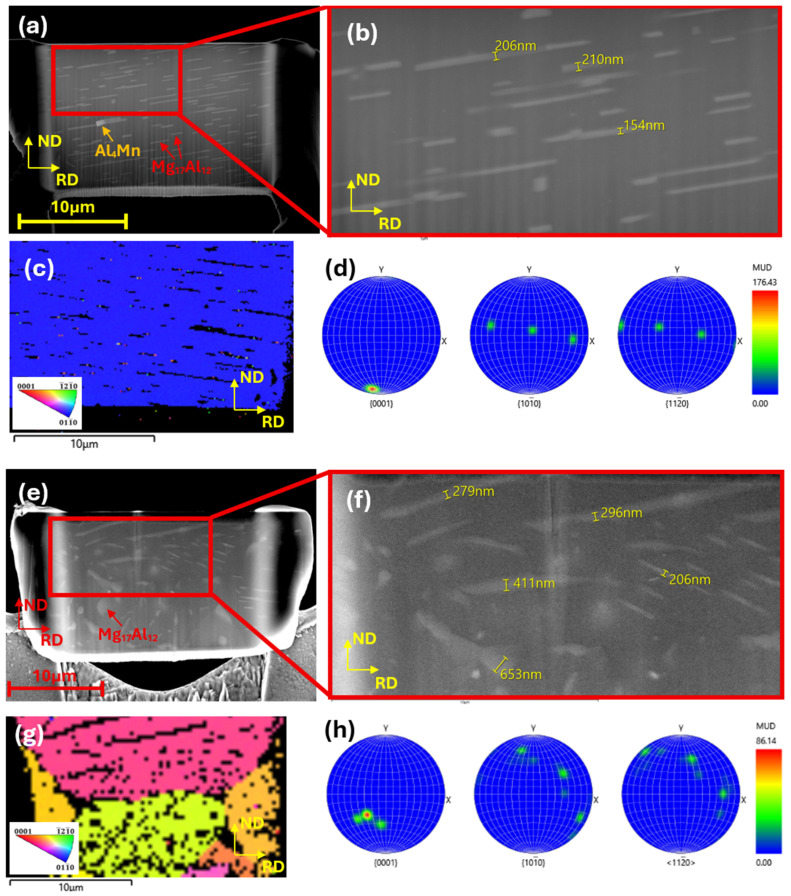
SEM, EDX, and EBSD analyses of the 131h-I and 131h-II specimens. (**a**) SEM image of the 131h-I specimen; (**b**) magnified view of (**a**); (**c**) EBSD orientation map corresponding to (**a**); (**d**) inverse pole figure (IPF) of the 131h-I specimen; (**e**) SEM image of the 131h-II specimen; (**f**) magnified view of (**e**); (**g**) EBSD orientation map corresponding to (**e**); (**h**) inverse pole figure (IPF) of the 131h-II specimen.

The microstructural features including crystallographic orientations of 131h-I and 131h-II were analyzed using transmission Kikuchi diffraction (TKD). The 131 h-aged specimen exhibited a high density of precipitates, consistent with the microstructural features observed in Figure 2d. The precipitates displayed a wide range of lengths, from less than 1 μm to more than 8.5 μm. In the case of 131h-I, the thickness of the Mg_17_Al_12_ precipitates was measured to be in the range of 154–210 nm (Figure 8b).

It is to note that the Mg_17_Al_12_ precipitates are nearly perpendicular to the ND, clearly seen in Figure 8. Because the 131h-I specimen is extracted from the grain having its c-axis almost parallel to the ND, this gives evidence that the Mg_17_Al_12_ precipitates are formed along the (0001) basal plane of the Mg matrix. Figure 8c,d show the IPF map obtained from TKD analysis and the grain orientation, respectively. From the pole figures, Figure 8d, it is clear that the long axis of the Mg_17_Al_12_ precipitates is aligned perpendicular to the c-axis of the matrix, i.e., parallel to the <10–10> and <11–20> axes. The long axis of the precipitate appears to rise from left to right in the SEM image, whereas it appears to be oriented downward in the TKD IPF map. The discrepancy between the SEM and TKD analyses arises from the observation of opposite sides of the FIB-prepared slice, resulting in a mirrored image of the microstructural orientation. The thickness of the Mg_17_Al_12_ precipitates in the 131h-II specimen is in the range of 206 nm to 653 nm, which is substantially thicker than the precipitates in Figure 8b. That is, the thickness of the Mg_17_Al_12_ phases formed on the (0001) basal plane varies with changing the observation plane. For example, if they are observed on a pyramidal plane the precipitates look thicker than in the case of observation from the prismatic plane. From the above results the formation of plate-shaped Mg_17_Al_12_ phase on (0001) plane is evident.

**Figure 9:**
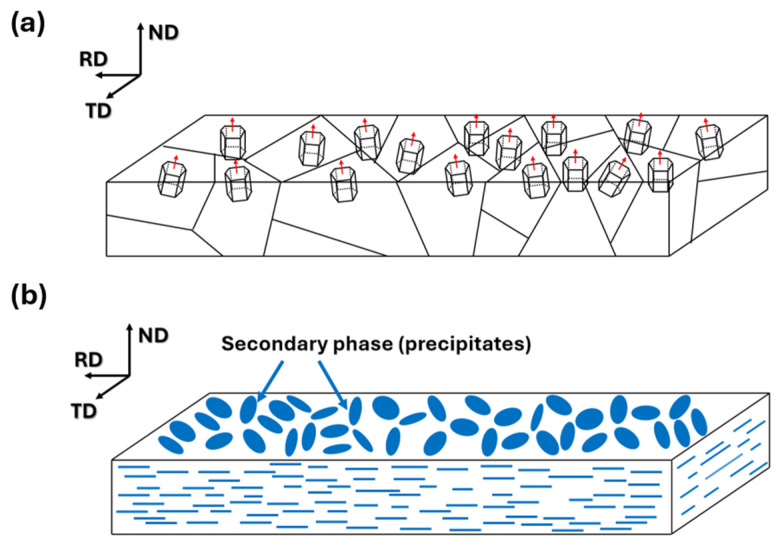
(**a**) Schematic illustration of the basal-type texture in the 131 h specimen. (**b**) Schematic illustration of the spatial alignment of Mg_17_Al_12_ precipitates in the 131 h specimen. The red arrows in (**a**) indicate the crystallographic c-axis of each grain.

A schematic representation indicating a strong basal-type texture of the 131 h specimen is shown in Figure 9a. Given this strong basal-type texture with the c-axes aligned parallel to the ND and the fact that the precipitates are oriented parallel to the (0002) plane, the alignment of Mg_17_Al_12_ precipitates can be schematically depicted as shown in Figure 9b. When incident electromagnetic waves penetrate the specimen perpendicular to the RD–TD plane, the plate-like Mg_17_Al_12_ phases act as secondary reflection sites, thereby enhancing the EMI SE [10].

In conclusion, the superior EMI SE of the 131 h specimen can be attributed to the increased volume fraction of Mg_17_Al_12_ precipitates with favorable orientation with the Mg matrix. These secondary phases enhance shielding performance by inducing multiple reflections of incident electromagnetic waves at the interfaces between the Mg matrix and the precipitate phase [8,10,14,15]. Consequently, the formation of dense and well-aligned precipitates is highly beneficial for improving EMI SE.

As shown in Figure 6 and Table 2, the 262 h specimen showed a lower EMI SE compared to the 131 h specimen. This degradation in shielding effectiveness can be attributed to the coarsening of the Mg_17_Al_12_ precipitates, as observed in Figure 4c,d. As already mentioned, 131 h specimen’s Mg_17_Al_12_ precipitate’s thickness ranged from 210 to 761 nm, whereas those in the 262 h specimen ranged from 328 to 836 nm. Such coarsening of the secondary phases accompanies the reduction in their length, which is also an effective way to reduce the interface energy between the Mg matrix and the Mg_17_Al_12_ phase. As a result, the multiple reflection of incident electromagnetic waves is diminished, leading to reduced EMI SE.

**Figure 10:**
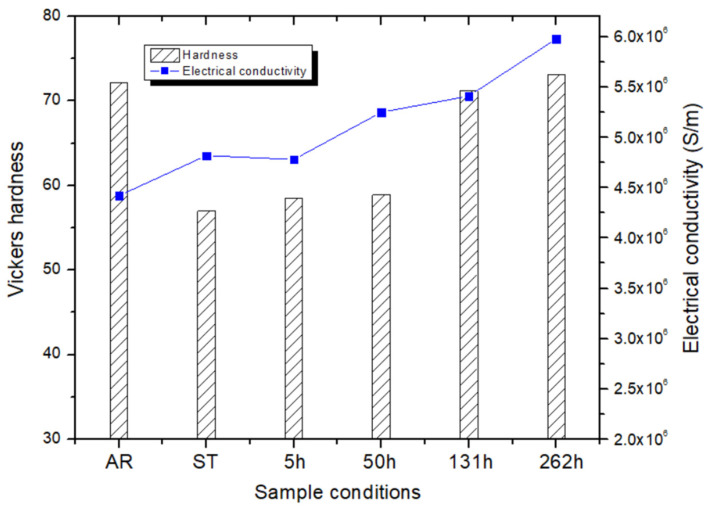
Vickers hardness of the AZ61 specimens under different sample conditions, measured under a load of 1 kgf and a dwell time of 10 s.

Figure 10 presents the Vickers hardness values of the AZ61 alloy at different sample conditions. The AR specimen has the highest dislocation density, resulting in a hardness of 72.2 HV. Since dislocation density is dramatically reduced by annealing, the ST specimen shows a decrease in hardness by 57.0 HV. The specimens aged for 5 h and 50 h exhibited hardness values comparable to that of the ST condition, indicating that age hardening in AZ61 is negligible up to 50 h.

After prolonged aging, the specimens aged for 131 h and 262 h exhibited increased hardness values of 71.2 HV and 73.1 HV, respectively, corresponding to increments of 12.3 HV and 14.2 HV compared with the 50 h specimen. Notably, this hardening response contrasts with the behavior of conventional age-hardenable alloys, where over-aging typically results in a decrease in hardness.

This anomalous hardening can be explained with reference to Figure 2. As shown in Figure 2c–e, the 131 h and 262 h specimens contain a markedly higher volume fraction of Mg_17_Al_12_ precipitates compared to the 50 h specimen. At these later stages of aging, the precipitates are densely distributed, and their interparticle spacing becomes smaller than the size of the Vickers indentation. As a result, the indenter inevitably presses the Mg_17_Al_12_ precipitates during hardness tests. Since Mg_17_Al_12_ precipitates is an intermetallic phase that is harder than the Mg matrix, this increment of the pressed Mg_17_Al_12_ phase during the operation of the Vickers hardness test contributes directly to the rise in measured hardness. Therefore, the rise in hardness observed from 50 h to 131 h and 262 h can be attributed to the substantially increased amount of Mg_17_Al_12_ phase during prolonged aging.

## 4. Conclusions

1. Electrical conductivity measurements of AZ61 specimens subjected to different processing conditions revealed that the as-rolled (AR) specimen exhibits the lowest conductivity. The conductivity increases after solution heat treatment and continues to increase with increasing aging time.
2. The AR and 131 h specimens exhibited the highest EMI SE even though the AR specimen showed the lowest electrical conductivity among other samples. The superior shielding performance of the AR specimen is attributed to its large amount of deformation-induced defects such as dislocations and twins, whereas the superior shielding performance of the 131 h specimen arises from an increased number of interfaces between the Mg matrix and Mg_17_Al_12_ precipitates with orientations favorable for enhancing EMI SE.
3. Specimens aged for more than 131 h developed a lamellar structure between the Mg matrix and the precipitates. The present study demonstrates that these precipitates are aligned parallel to the (0002) plane of the Mg matrix, and such orientation is beneficial for improving the EMI SE of the AZ61 alloy.
4. The AZ61 alloy aged at 250 °C exhibited negligible hardening up to 50 h, while the 262 h-aged specimen retained the highest hardness despite an over-aged microstructure. This unusual hardening behavior can be attributed to the high density of Mg_17_Al_12_ precipitates formed during prolonged aging, which reduces the interparticle spacing and increases the probability of indenter pressing the hard Mg_17_Al_12_ phase during hardness testing.

## Figures and Tables

**Table 1:** Specimen designations according to the undergone processes.

Sample Condition	Designation
Hot rolled	AR
Hot rolled + Solution treatment (400 °C, 18 h)	ST
Hot rolled + Solution treatment (400 °C, 18 h) + Aging (250 °C, 5 h)	5 h
Hot rolled + Solution treatment (400 °C, 18 h) + Aging (250 °C, 50 h)	50 h
Hot rolled + Solution treatment (400 °C, 18 h) + Aging (250 °C, 131 h)	131 h
Hot rolled + Solution treatment (400 °C, 18 h) + Aging (250 °C, 262 h)	262 h

**Table 2:** Electromagnetic interference (EMI) shielding effectiveness (SE) of AZ61 specimens measured at frequencies of 500, 900, and 1200 MHz, along with the mean EMI SE values.

Sample Condition	Designation					
	AR	ST	5 h	50 h	131 h	262 h
EMI SE at 500 MHz	83.8	84.7	73.7	83.6	86.1	85.3
EMI SE at 900 MHz	83.9	81.2	53.0	71.1	83.4	77.4
EMI SE at 1.2 GHz	82.5	73.8	49.2	70.3	81.9	72.7
Mean EMI SE (from 30 MHz to 1.5 GHz)	77.0	74.6	58.7	71.2	77.8	73.2

**Table 3:** Electrical conductivity of AZ61 alloy samples processed under different conditions.

Sample Condition (Designation)	Average Conductivity (S/m)
As-Rolled (AR)	$4.421\times 10^{6}$
Solution heat-treated (ST)	$4.818\times 10^{6}$
Aged 5 h (5 h)	$4.780\times 10^{6}$
Aged 50 h (50 h)	$5.250\times 10^{6}$
Aged 131 h (131 h)	$5.409\times 10^{6}$
Aged 262 h (262 h)	$5.979\times 10^{6}$

## Data Availability

The original contributions presented in this study are included in the article. Further inquiries can be directed to the corresponding authors.

## Abbreviations

The following abbreviations are used in this manuscript:

EMI	Electromagnetic interference
SE	shielding effectiveness
OM	Optical microscope
SEM	Scanning electron microscope
EDX	Energy-dispersive X-ray spectroscopy
EBSD	Electron backscatter diffraction
FIB	Focused ion beam
XRD	X-ray diffraction
AR	As-rolled
ST	Solution treatment
RD	Rolling direction
ND	Normal direction
TD	Transverse direction
IPF	Inverse pole figure
TKD	Transmission Kikuchi diffraction
${SE}_{tot}$	Total shielding effect
${SE}_{R}$	Reflection contribution to shielding effect
${SE}_{A}$	Absorption contribution to shielding effect
${SE}_{B}$	Multiple reflection contribution to shielding effect
f	Frequency of electromagnetic radiation
$\mu_{r}$	Relative magnetic permeability
μ	Magnetic permeability
$\sigma_{r}$	Relative electrical conductivity
σ	Electrical conductivity
t	Shield thickness
